# The Role of Biostimulants as Alleviators of Biotic and Abiotic Stresses in Grapevine: A Review

**DOI:** 10.3390/plants11030396

**Published:** 2022-01-31

**Authors:** Eliana Monteiro, Berta Gonçalves, Isabel Cortez, Isaura Castro

**Affiliations:** 1 Centre for the Research and Technology of Agro-Environmental and Biological Sciences (CITAB), University of Trás-os-Montes e Alto Douro (UTAD), 5000-801 Vila Real, Portugal; bertag@utad.pt (B.G.); icortez@utad.pt (I.C.); icastro@utad.pt (I.C.); 2 Institute for Innovation, Capacity Building and Sustainability of Agri-food Production (Inov4Agro), University of Trás-os-Montes e Alto Douro (UTAD), 5000-801 Vila Real, Portugal; 3 Department of Biology and Environment, University of Trás-os-Montes e Alto Douro (UTAD), 5000-801 Vila Real, Portugal; 4 Department of Agronomy, University of Trás-os-Montes e Alto Douro (UTAD), 5000-801 Vila Real, Portugal; 5 Department of Genetics and Biotechnology, University of Trás-os-Montes e Alto Douro (UTAD), 5000-801 Vila Real, Portugal

**Keywords:** *Vitis vinifera* L., sustainability, climate change, biotic and abiotic stresses, plant fungal diseases

## Abstract

The viticulture and wine industry contribute to the economy and reputation of many countries all over the world. With the predicted climate change, a negative impact on grapevine physiology, growth, production, and quality of berries is expected. On the other hand, the impact of these changes in phytopathogenic fungi development, survival rates, and host susceptibility is unpredictable. Grapevine fungal diseases control has been a great challenge to winegrowers worldwide. The use of chemicals in viticulture is high, which can result in the development of pathogen resistance, increasingly raising concerns regarding residues in wine and effects on human and environmental health. Promoting sustainable patterns of production is one of the overarching objectives and essential requirements for sustainable development. Alternative holistic approaches, such as those making use of biostimulants, are emerging in order to reduce the consequences of biotic and abiotic stresses in the grapevine, namely preventing grape fungal diseases, improving grapevine resistance to water stress, and increasing yield and berry quality.

## 1. Introduction

Grapevine (*Vitis vinifera* L.), a perennial woody plant, constitutes the most valued fruit species globally and has been linked to agricultural and religious activities [1,2]. Most parts of the grapevine are used, principally berries, for manufacturing various industrial products, such as, wine, raisins, pressed juice, and spirits [3,4,5]. The winemaking sector has a socio-economic relevance in many countries, contributing to the exports and sustaining many wine-related activities, including tourism. In 2020, five countries represented 50% of the world vineyard surface area, namely: Spain (13.1%), France (10.9%), China (10.7%), Italy (9.8%), and Turkey (5.9%) [6]. According to OIV, in the same year the European countries were the biggest wine producers, namely, Italy (49.1 mhl), France (46.6 mhl) and Spain (40.7 mhl), followed by the USA (22.8 mhl) and Argentina (10.8 mhl) [6]. It is known that biotic and abiotic stresses can limit the growth and also the yield of plants [7]. In the global climate change scenario, viticulture faces new challenges and threats. The winegrowing regions are restricted at geographic and climatic level, which is synonymous of quality and optimum production [8]. These regions present a specific “terroir”, which includes specific soil, topography, climate, landscape characteristics and biodiversity features, and interaction with applied vitivinicultural practices [9,10]. The climate change impacts in grapevine are visible in phenology, yield, wine quality and will increase the pressure of pests and diseases in the vineyards, due to the milder winters [11,12]. One strategy that has become interesting is the foliar application of biostimulants to prevent plant diseases and improve berry quality on the grapevine. The foliar application of biostimulants acts as plant biostimulants enhancing plant growth and nutrient uptake, being also an alternative to soil fertilization, avoiding some of the negative effects to the environment from leaching of nutrients into the groundwater. The application of these natural compounds has effects on plant physiology, pathogens development and leads to a diverse expression of plant genes responsible for triggering metabolic pathways and plant defense responses [13].

This review will update the state of the art on this topic, addressing several research studies that discuss the best compounds in different species/cultivars and at the same time explaining their mode of action [7,13,14,15,16,17,18,19,20,21]. Here we cover the most used biostimulants in the vineyard, highlighted towards environment-friendly viticultural practices.

## 2. Impacts of Climate Change on Grapevine

Climate change poses new challenges and threats for viticulture since the quality of grapes and consequently wine depends on several climatic factors. The main factors that have this influence are water status, radiation, temperature, and greenhouse gases (CO_2_) [22,23]. The water status of grapevine depends on soil texture, percentage of stones, rainfall, evapotranspiration, rooting depth, and leaf area [22]. Water stress impairs photosynthesis, shoot growth, decreases berries size, increases grape tannin and anthocyanin contents, with effective changes in wine aroma. On the contrary, excessive humidity during the early stages overstimulates vegetative growth, which leads to denser canopies and a higher likelihood of disease problems in leaves and in inflorescences [24]. The radiation is a factor that is difficult to separate from the effect of temperature. This can increase the photosynthetic rate (without water stress), and high UVB irradiation can enhance the color and flavor of wine and in the case of red grapes may increase the tannin synthesis [22]. However, the combination of excessive radiation and temperature can lead to sunburn in leaves and in berries. This can be exacerbated by other stress factors, such as water deficit, and cause significant losses in the quality and yield of wine grapes [25]. In relation to greenhouses gases, the increase in CO_2_ will influence the growth of the grapevine and the quality of the grapes. In fact, high CO_2_ concentration in the atmosphere can increase the photosynthetic rate, vegetative growth, and water use efficiency, thus leading to higher yield [23]. In future decades, it is expected that the global mean surface temperature increases at about 0.2 °C per decade, reaching values between 1 °C and 6 °C at the end of the XXI century according to all reports on emissions scenarios, which poses a huge problem for viticulture [23,24], the temperature being the factor that most affects grapevine phenology. The morphological and physiological changes are responsible for the vegetative and reproductive cycles of grapevine, which comprises two phases: (1) the growing season, where the plant is active with a constant change in plant morphology; and (2) the dormancy, where its external appearance is maintained and the physiological activity is very reduced [8,24]. The duration of each phase differs according to the grapevine variety and is dependent on the thermic conditions of each region [24]. Future likely climate change scenarios will influence the regular course of the phenological stages of the grapevine [26]. Abiotic stress, in addition, affect plant growth and development, crop production quality and yield, and can be really compromising if it occurs in the most sensitive phenological phases of plants [27]. Temperatures, high irradiation, and soil water storage affect vine growth and wine production in many ways [28]: (i) during the dormant stage, the minimum temperature or effective chilling units (hours below a certain temperature, normally bellow 10 °C) are needed to ensure uniform budbreak; (ii) during spring, vegetative growth of grapevines is initiated by prolonged temperatures above 10 °C; (iii) during anthesis and berry development, prolonged days of temperature greater than 30 °C can induce heat stress in the vine, a premature veraison (changes in berries color and accumulation of sugars that increase the alcohol content in wine), increase plant mortality, berry abscission, and enzyme inactivation, and reduce flavor development in the fruit; and (iv) during the maturation stage, a pronounced diurnal temperature range enhances the synthesis of tannins, sugars, and flavors; the grape acidity can be reduced, because the malic acid content decreases with high temperatures [22,24,28].

On the other hand, a grapevine is susceptible to various pathogens, fungal diseases being the major risk that compromises their cultivation and economic profit worldwide [29,30]. The most problematic fungi and fungi-like pathogens to *Vitis vinifera* are: downy mildew (*Plasmopara viticola*), powdery mildew (*Erysiphe necator*) and gray mold (*Botrytis cinerea*) [31,32,33]. These infections reduce fruit quality and yield, either by direct infection of berries themselves or through a decrease in plant vigor [33]. Many synthetic chemical pesticides are used intensively and became indispensable for traditional agriculture, preventing, and limiting pathogen infections. It is known that viticulture is one of the most treated cropping systems in the world; in wine-growing regions fungicides can account for more than 90% of all pesticide applications [29,30]. To control downy mildew the winegrowers frequently use products with copper, namely in organic production [34]. Due to the continuing use of copper and the fact that it can induce phytotoxicity in the grapevines and lead to an accumulation of this heavy metal in vineyard topsoil, the European Commission has taken some restrictive measures in the use of copper-based formulations. Moreover, the use of the other fungicides is limited. The restrictions applied by the European Commission are on the number of pesticide treatments (Directive 2009/128/EC) and on the maximum quantity of copper fungicides per year (Regulation 2002/473/EC). Copper has been added to the list of candidates for substitution (European Commission Implementing Regulation 2018/84) and, since February 2019, further limited to 4 kg per hectare/year spread over 7 years (European Commission Implementing Regulation 2018/1981 of 13 December 2018) [35]. In parallel, there is a demand by consumers for more environment-friendly products. Consequently, research in innovative bio-strategies is necessary. Recently, a study contributed with an innovative approach to obtain specific protection against the causal agents of grape downy mildew (*P*. *viticola*) [36]. In this study, the authors used the yeast two-hybrid approach and the *P*. *viticola* cellulose synthase 2 (PvCesA2) as target enzyme to identify interacting peptides, potentially capable of inhibiting this enzyme. Thus, they demonstrated that the peptide NoPv1 prevents *P*. *viticola* germ tube formation and grapevine leaf infection without affecting the growth of non-target organisms and without being toxic to human cells. This approach may bring many benefits in the future due to its specificity and because *P*. *viticola* is one of the biggest problems for viticulture.

## 3. Use of Biostimulants as a Mitigation Strategy for Biotic and Abiotic Stresses in Grapevine

A biostimulant is a formulated product containing mixtures of natural substances and/or microorganisms applied to plants with the aim to enhance nutrition efficiency, abiotic and biotic stress tolerance, and/or crop yield and quality traits, regardless of its nutrients content, plant growth regulators, or plant protective compounds [20]. In general, nine categories of substances that act as biostimulants can be defined: (i) humic substances; (ii) complex organic materials (obtained from agro-industrial and urban waste products, sewage sludge extracts, composts, and manure); (iii) beneficial chemical elements (Al, Co, Na, Se, and Si), (iv) inorganic salts including phosphite; (v) seaweed extracts (brown, red, and green macroalgae); (vi) chitin and chitosan derivates; (vii) antitranspirants (kaolin and polyacrylamide); (viii) free amino acids and N-containing substances (peptides, polyamines, and betaines); and (ix) plant growth-promoting rhizobacteria (PGPR), arbuscular mycorrhizal fungi (AMF) and *Trichoderma* spp. [37]. Due to the complexity of the extracts and the wide range of molecules contained in the solutions, it is very difficult to understand which are the active compounds [38]. Biostimulants are usually able to improve vigor, stimulate vegetative growth, improve nutrient uptake and distribution within the plant, increase the antioxidant capacity of plant tissues and enhance tolerance to biotic and abiotic stress, consequently improving plant yield and fruit quality [27,39]. These products contain some bioactive molecules called elicitors, which have a beneficial effect on plants and improve their ability to face adverse environmental conditions, acting on primary or secondary metabolism [18]. The term “elicitor” refers to all the signal molecules that are perceived and that induce a defensive reaction in the plant [16]. It has been reported that exogenous application of elicitors, can induce the activation of enzymes involved in the synthesis of phenolic compounds and, consequently, can play a key role in plant-pathogen interactions [18,29]. In the case of grapevine, diseases usually take an excessive application of fungicides that have several negative impacts, such as development of fungicide resistance; accumulation of fungicide compounds in the vineyards topsoil; ecological consequences on soil, water, fauna, and risks to human health [34,40,41]. In addition, pesticide residues have been identified in the wine, affecting the natural yeast communities necessary for winemaking, as well as its aroma [29]. With the need to preserve the quality of wine and to reduce the impact of pesticides on the environment and human health, considerable interest has been focused on replacing chemicals with innovative bio-strategies, such as environment-friendly products, to promote sustainable agriculture and food production systems. Thus, the use of biostimulants as biofungicides is a promising alternative for the control and prevention of fungal and fungal-like diseases. They are currently considered an emerging class of crop management products that aim to moderate crop stress in order to increase crops productivity. Over the last decade, utilization of natural plant biostimulants is gaining importance, and these plant extracts have been analyzed in several studies in different crops, in order to reduce the use of chemicals [42,43,44,45,46]. The use of plant extracts as biostimulants gives importance to plants that are normally undervalued, without any commercial interest. Thus, these plants become useful to improve the performance of plants and at the same time to control fungal diseases in grapevine and other plant species.

## 4. Biostimulants Used in Grapevine

**Figure 1:**
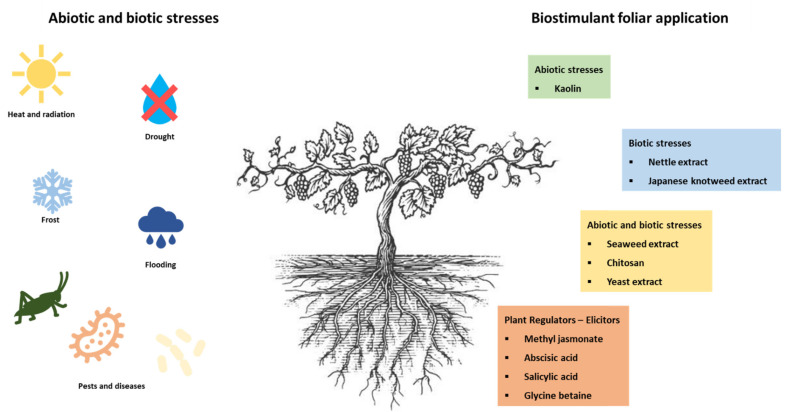
Different biostimulants used as stress alleviators in grapevine.

Winegrowers have the need to preserve the quality of the grapes and consequently of the wine and face a great challenge due to climate change that increases biotic and abiotic stress. At the same time, it is necessary to make viticulture more sustainable and environment-friendly, reducing the use of chemical products. There are several innovative bio-strategies already used, as is the case with biostimulants. Some of the most frequently used in the vineyard are shown in Figure 1. The different biostimulants were grouped according to the stress they fight, biotic, abiotic, or both and those that act as elicitors. Some of these biostimulants are plant extracts, namely nettle, Japanese Knotweed, and seaweed extracts. There are other products, such as yeast extracts, urea, kaolin, and others, which are also beginning to be interesting in combating stress in the vine caused mainly by climate changes.

### 4.1. Biostimulants as Abiotic Stress Alleviators 

#### Kaolin

Kaolin is a chemically inert and non-toxic clay that can reflect radiation [47]. This compound is widely used as a mitigation strategy to handle summer stress, namely water stress, excessive radiation that is absorbed by leaves and grape berry clusters, elevated air temperature, and high evaporative demand [2,10]. Foliar application of this clay mineral has become commonly used in Portuguese vineyards and is very common in Douro Demarcated Region (DDR) [2,47,48]. Some studies report some concerns about this compound because it forms a particle film that can affect the photosynthetic rate [2], while other studies proved otherwise the stimulation of the photosynthetic activity (Table 1) [47]. Indeed, the study of Dinis et al. [47] proved that the reflective film caused by kaolin application can protect the photosystem II structure. This study highlights the impact of kaolin application on the photosynthetic activity of grapevine in different growing conditions (light and irrigation), which provides new insights for the application of this compound towards the adaption of the grapevines to different climate conditions.

### 4.2. Biostimulants as Biotic Stress Alleviators

#### 4.2.1. Nettle

Nettle (*Urtica dioica*) is an herbaceous perennial flowering plant that belongs to the Urticaceae family and *Urtica* genus and is used in a large diversity of agronomic crops, as fertilizer, as forage, or as livestock feed [72]. It is native to Eurasia and occurs as a perennial plant in temperate zones of Asia, America, and Europe [72,73]. Various parts of the nettle have been used for human welfare; it is historically used as a medicinal plant and has great economic potential due to its multi-utilitarian nature [72,74]. It is popularly cooked green in many regions due to its high protein content, 21–23% crude protein, and 9–21% crude fiber [73]. Nettle is easily digestible and has a high content of minerals and vitamins, especially iron, manganese, potassium, calcium, vitamin C, D, and pro-vitamin A [72,73,75]. The two most prevalent active chemical agents found in nettle are formic acid and histamine, which function as anti-inflammatory agents [73]. In biodynamic viticulture, an alternative agricultural technique, nettle is widely used in biodynamic preparations [76,77,78,79]. Some studies also show that nettle is efficient as an antifungal against different genera and species of fungi, namely *Alternaria alternata* and *Rhizoctonia solani* [74]; *Curvularia lunata*, *Alternaria solani*, *Alternaria zinniae*, *Fusarium oxysporum* [80]; *Phytophthora* spp. [43] and *Botrytis cinerea* [46]. In the future, more studies are needed on the use of nettle extracts on the grapevine, as these extracts seem promising in combating and preventing fungal diseases.

#### 4.2.2. Japanese Knotweed

Japanese knotweed (*Fallopia japonica*) is an invasive plant native from Asia, namely, Korea, China, and Taiwan [81,82,83]. This is a woody-stemmed herbaceous perennial rhizomatous plant, member of the buckwheat (Polygonaceae) family and has different scientific names, *Reynoutria japonica*, *Polygonum cuspidatum,* and the most used *Fallopia japonica* [82,84,85]. *Fallopia japonica* was introduced in Europe and North America more than 100 years ago as a source of food and as an ornamental plant [85,86,87]. Japanese knotweed is able to produce high concentrations of secondary metabolites (stilbenes, tannins, lignin, anthocyanins, sterols, phenethyl alcohols, and essential oils), anthraquinones being the most important [83,85]. At the agronomical level, anthraquinones have a lot of applications: as a repellent from pests, namely to combat mosquitos [88]; in sunflowers, to protect the seeds against blackbirds [89]; and as an antifungal in grapevine [45] (Table 1).

### 4.3. Biostimulants as Abiotic and Biotic Stress Alleviators

#### 4.3.1. Seaweed Extracts

The use of seaweed formulations as biostimulants has been reported for many years and is well established [90,91]. Macroalgae form an integral part of marine coastal ecosystems. It has been estimated that there are about 9000 macroalgae species [92]. They are classified into three main groups based on their pigmentation: brown (Phaeophyta), red (Rhodophyta), and green algae (Chlorophyta) [92]. Many seaweed species are an underutilized bioresource, often used as a source of food, industrial raw materials, and in therapeutic and botanical applications, in agriculture and horticulture to feed livestock, for soil fertilization, and in the form of extracts to promote plant growth [90,92]. Seaweeds contain several macro and micronutrients, vitamins, amino acids, cytokinins, auxins, and abscisic acid [91,92]. At the agronomical level, a number of commercial seaweed extract products are available, and several studies have shown their good results [92]. Brown seaweeds are the most used type in agriculture, *Ascophyllum nodosum* L. being the most studied [49,50,93,94,95,96,97,98,99,100]. Some studies in *Vitis vinifera* have shown that foliar application is beneficial to control and prevent the impacts of biotic and abiotic stress (see Table 1).

#### 4.3.2. Chitosan

Chitosan has antimicrobial properties and is able to elicit plant defense to pathogens [17]. This compound is used in grapevine to control fungal diseases [34,53,54,55]. Chitosan is a beta-1,4-linked glucosamine, a deacetylated derivative of chitin, present in the shells of crustaceans, in insects and in certain organisms, such as fungi, algae, and yeast [29,53,58]. It is a polymer that forms a semipermeable film around plant tissues, inhibiting several pathogens and induces defense response mechanisms in the host tissues [34]. Several studies demonstrate the use of this compound in the vineyard and the capacity to improve the grapevine defense responses and also the grape production and quality under stress (Table 1). The effectiveness of treatment depends on its concentration and, the stage of plant development at a time when they were exposed to the drought or heat stress [58]. In the future, the use of chitosan as a biostimulant, may be useful to prevent and control some of the most important fungal-like diseases in grapevine, with the benefits for the consumers that are concerned about the presence of fungicide residues in berries and wines. This biostimulant is a good candidate for the substitution of products with copper frequently use by winegrowers, since it is a natural compound, safe for humans and the environment, its use is a strategy welcomed by organic growers and can be applied to manage grapevine diseases [55].

#### 4.3.3. Yeast Extracts

Yeast has been used for many years in several processes, namely fermentation and food industry (production of alcoholic beverages, biomass), production of various metabolic products (enzymes, vitamins, capsular polysaccharides, carotenoids, polyhydric alcohols, lipids, glycolipids, citric acid, ethanol, carbon dioxide), medical science, research, and agriculture [101,102]. It is known that yeast cell walls are rich in mannoproteins, ß-1,3-glucan, ß-1,6-glucan, and chitin, while the plasmatic membrane comprises lipids, sterols, and proteins [103]. The yeast extracts contain several compounds that may act as elicitors [17,18]. Yeast extracts have been used in grapevine with the aims such as improving the synthesis of phenolic amino acids and, volatile compounds in berries and, subsequently, in the wines [18]. Several studies using yeast extracts compare its effectiveness to that of other compounds, such as methyl jasmonate, chitosan, and seaweeds [17,34,62,104,105]. Yeast extracts (*Saccharomyces cerevisiae*) have been used in cv. Tempranillo retrieving better results when compared with methyl jasmonate and chitosan in terms of berry and wine anthocyanin content [17]. Several authors reported different inter-annual and inter-varietal responses in grapevine to the foliar applications of yeast extracts. In a study using yeast cell wall, as preharvest treatments, in the cvs. Monastrell and Tempranillo, annual and varietal differences in stilbene content were detected [62]; in another study inter-varietal differences in the amino-acid contents were detected in the cvs. Garnacha, Graciano and Tempranillo sprayed with yeast extracts [104]. On the other hand, the same authors reported that the application of yeast extracts has a physiological cost for the vines, leading to a decrease in the content of free and total amino acids in the cv. Tempranillo, although inducing resistance to diseases. Other studies with these extracts in grapes and wines are presented in Table 1. Yeast extract applications in grapevine have many benefits, but the consequences in physiological parameters of the plant must be considered. Considering this, in the future it may be a simple practice to increase the phenolic content of grapes and wine and to combat and prevent fungal diseases. However, further studies are needed to determine which cultivars benefit the most from using yeast extracts as biostimulants in the vineyards.

### 4.4. Biostimulants as Plant Regulators and Elicitors 

#### 4.4.1. Methyl Jasmonate

Methyl jasmonate (MeJA) is a derivative of jasmonic acid (JA), known to be a ubiquitous plant signaling compound and was primarily isolated from the essential oil of *Jasminum grandiflorum* [65]. Methyl jasmonate is an endogenous plant regulator, that acts as a signaling molecule upon plant stress and can be involved in mechanisms of plant defense by the synthesis of secondary compounds [17]. MeJA is one the most used elicitors on grapevine, that can induce defense mechanisms [18]. Increase of transcript levels coding pathogenesis-related proteins (PR proteins), coding enzymes involved in phytoalexin biosynthesis, have been verified and correlated with the accumulation of stilbenes (antimicrobial compounds) [33]. This study confirmed the eliciting activity of MeJA. Several studies also report the exogenous application of MeJa as inducer of berry and wine quality (Table 1). Application of this compound may be a simple and innovative strategy to improve the physicochemical and physiological parameters of grapevine, increasing berries and wine quality. In the future more studies are needed to prove the elicitor efficiency of methyl jasmonate against grapevine pathogens.

#### 4.4.2. Abscisic Acid

Abscisic acid (ABA) is an elicitor extracted from plants and is used in grapevines as a growth regulator [18,63,65]. ABA protects plants from environmental stress and has a positive regulatory effect on plant growth [65,106]. ABA can induce responses to water, light, and thermal stresses [106]. ABA can be involved in the transformation of sugar and organic acids in berries (Table 1) [65] and can also enhance the expression of key genes (*Phenylalanine Ammonia Lyase* (*PAL*), *Chalcone Isomerase* (*CHI*), and *Myeloblastosis transcription factors* (*MYB*)) in the synthesis pathway of polyphenols and anthocyanins. This hormone can be used as a biostimulant to improve grape and wine quality.

#### 4.4.3. Salicylic Acid

Salicylic acid (SA) plays an important role in plant development, enhances plant vigor under biotic and abiotic stresses, and is one of the endogenous signals involved in mediated responses associated with resistance to biotrophic pathogens [14,107,108]. Several studies have shown that the exogenous application of SA enhances the growth and productivity of plants [108]. In cherries, it was used to prevent cracking [98]; in olive trees—to protect from drought [109]; and in strawberries—to improve fruit quality [110]. In the case of grapevine (Table 1), SA has been demonstrated as an important secondary metabolite playing an essential role in determining berry quality parameters such as color, flavor, astringency, and bitterness [66]. Despite all the advantages that this compound seems to present, it is known that exogenously applied SA may have a negative effect on photosynthetic activity [15]. Like most other classes of plant hormones, the application of SA at levels above the ideal can cause inhibitory effects on the growth, physiological or metabolic processes of plant tissues; at lower levels (close to the ideal) it can often have beneficial effects on these processes, especially in stressed plants [15]. In the future, the exogenous application of SA to vineyards would be an interesting agronomic practice for obtaining grapes with improved properties. However, the effect may depend on the combination of several factors, including genotype, type/level of stress and the concentration applied.

#### 4.4.4. Glycine Betaine

Glycine betaine (GB) is an N-trimethyl glycine derivative compound that belongs to the class of quaternary amines and can be found in a wide range of bacterial, plant, and animal species [111,112]. GB has several properties, attractive for use in stress protection, since it is naturally synthesized, readily available, non-toxic, inexpensive, and maintains water content in plant cells by lowering osmotic potential in osmotic adjustment [111,113,114,115]. This is a convenient compound for the induction of crop tolerance to various abiotic stressors, such as frost, extreme temperatures, or drought [112,113,116]. There is evidence suggesting that GB plays a role in response to stresses in some plant species, where the accumulation occurs mainly in chloroplasts to protect the thylakoid membrane [113]. This product retains high photosynthetic capacity, promoted plant growth, maintained the yield, and enhanced performance under stress [15]. The exogenous application of GB was used in different crops, such as sweet potato [117], alfalfa and cowpea [118], strawberry [112], and cherry [119]. The application of glycine betaine to grapevines at critical periods (e.g., prior to spring frosts) may protect plants and maintain yields [113]. However, high concentrations of glycine betaine can result in severe phytotoxicity for plants, and the concentrations for each plant species must be determined [112,113]. In grapevine, it is known that concentrations higher than 50 mM result in severe phytotoxicity [113]. Some studies have been developed regarding the application of glycine betaine in grapevines under drought. The effect of foliar applications of GB (15 mM) has been studied at four growth stages (before flowering, flowering, bunch closure, and veraison) under water stress (plants irrigated by 70% of the required water) [60] (Table 1). In a recent study in cherry, it was found that the foliar application of GB leads to an increase in the cuticle thickness [98]. These results are very promising for the application of glycine betaine in grapevine as the increase in the thickness of the cuticle may protect the berries against water loss and infection by fungi and also by pests. The application of GB in grapevine might be common in the near future since this compound can be easily acquired by farmers as it is a low-cost product.

## 5. Grapevine Responses to Biostimulants

Biostimulants can have three different modes of action: plant growth promoters/inhibitors, stress alleviators, and combined action [120]. These modes of action are dependent on the different bioactive compounds present in the biostimulants, of which little is known. Biostimulants can improve plant performance acting directly on the plant physiology and metabolism or by improving the soil conditions [121]. On the other hand, biostimulants can also act as elicitors, acting as stress alleviators, involved in resistance to drought, salinity, and in mechanisms of response to pathogens. The plant responses to biostimulant applications have been usually associated with the signaling of bioactive molecules in the primary and secondary metabolisms [120]. While the effects of these biostimulants are documented in several species, little is known about the mechanisms of action, due to the variable and complex nature of these substances. The beneficial effects of biostimulant applications can be associated with several biochemical and physiological mechanisms. In grapevine, the application of these compounds triggers responses in the cellular membrane, chloroplast, and nucleus protecting this species against biotic and abiotic stresses. 

**Figure 2:**
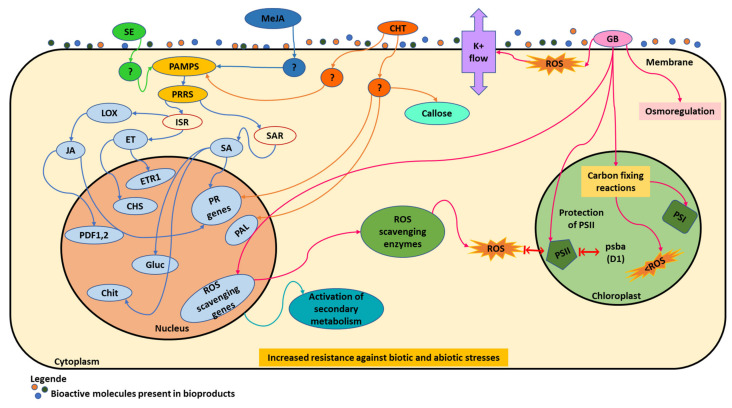
Schematic representation of the modulation on some pathways in grapevine by biostimulants.

A summary of some mechanisms that can be triggered by the different biomolecules present in the biostimulants is shown in Figure 2. Some biostimulants act as elicitors, such as seaweed extracts (SE, green) and chitosan (CHT, orange); or as plant-derived elicitors, such as methyl jasmonate (MeJA, blue). Figure 2 schematically shows how these molecules act as elicitors. First, they act as pathogen-associated molecular patterns (PAMPs), which bind to host transmembrane pattern recognition receptors (PRRs). Then, plants are prepared for the induced systemic resistance (ISR) that occurs naturally, the defensive capacity being developed by a plant when appropriately stimulated. They also activate the systemic acquired resistance (SAR), characterized by an accumulation of salicylic acid (SA) and PR proteins. In the case of an ISR mechanism, jasmonic acid (JA) and ethylene (ET) pathways are important for the induction of broad-spectrum disease resistance. In fact, some authors verified that responses to *B*. *cinerea* attack in grapes are mediated by jasmonic acid and ethylene; while in SAR, accumulation of salicylic acid (SA) occurs both locally and, at low levels, systemically [107]. Salicylic acid plays a crucial role in the induction of several families of pathogenesis-related genes, such as β-1,3-glucanases and chitinases, capable of hydrolyzing fungal cell walls [14] (Table 2). Plants sprayed with these biostimulants react quickly and plant cell membrane receptors bind to elicitor molecules, induce local resistance, and subsequently generate plant molecular responses. This fact can be explained because some biostimulants can be derived from plants that acquire resistance for their own diseases and now can act as elicitors when applied in other crops, such as grapevine. 

Another mechanism in Figure 2 refers to glycine betaine (GB, pink), which protects plants mostly against oxidative stress, which is caused by several adverse conditions. This generally occurs, when the balance between the production of reactive oxygen species (ROS) and the quenching activity is perturbed by an external stressor [27]. It is known that ROS are normally produced at low levels by various metabolic processes, such as photosynthesis or respiration, playing an important role in signaling related to the growth and development of plants [27]. Under stress conditions, the amount of ROS increases dramatically, which can result in cell damage, death, and toxicity to proteins, lipids, or nucleic acids. Through the activity of the antioxidant system, ROS concentration is maintained at non-toxic levels [27]. Biostimulants can activate antioxidative enzymatic function and increase ROS scavenging enzymes that are required to inactivate toxic free oxygen radicals produced in plants under stress, namely drought and salinity [15,27]. Glycine betaine can act in six different ways [15]: (1) GB limits efflux of K^+^ ions induced by ROS, with the protection of membrane integrity or by a channel-blocking function; (2) this compound can protect the transcription machinery activating the genes for ROS-scavenging enzymes, reducing the ROS levels and the effects of abiotic stress in photosynthetic machinery; (3) the protection of the photodamaged PSII is induced by *psbA* gene that encodes the D1 protein; (4) GB participation in osmoregulation and in the protection of biological membranes integrity against the effects of abiotic stresses and ROS; (5) when GB biosynthesis consumes photosynthesis-generated electrons, may alleviate electron transport chain, which prevents overreduction of the photosynthetic electron transport chain, thus lowering the probability for generation of ROS; (6) the protection of the integrity of carbon-fixing enzymes, maintaining the higher rates of CO_2_ fixation (PSI), even under stress.

Biostimulants can act as elicitors, such as seaweed extracts (green) and chitosan (orange), or as a plant-derived elicitor, such as methyl jasmonate (blue), when they act as pathogen-associated molecular patterns (yellow). Glycine betaine (pink), can act in six different ways, protects plants mostly against oxidative stress, that is caused by several adverse conditions. Seaweed extracts (SE); methyl jasmonate (MeJA); pathogen-associated molecular patterns (PAMPs); pattern recognition receptors (PRRS); induced systemic resistance (ISR); lysyl oxidase (*LOX*); jasmonic acid (JA); promoter-deletion 1,2 (PDF 1,2); pathogenesis-related (PR) genes; ethylene (ET); ethylene response 1 (ETR1); chalcone synthase (CHS); systemic acquired resistance (SAR); salicylic acid (SA); β-1,3-glucanase (Gluc); chitinase (Chit); chitosan (CHT); phenylalanine ammonia lyase (PAL); glycine betaine (GB); reactive oxygen species (ROS); photosystem I (PSI); photosystem II (PSII); *psbA* (Photosystem II protein D1 precursor).

### Differential Expression of Genes Involved in Metabolic Grapevine Pathways upon Biostimulants Application

Several studies have been carried out to verify the molecular regulation and gene expression triggered by biostimulant applications in grapevine (see Table 2). Defense-related genes and those involved in the regulation of secondary metabolism are among the most studied. On grapevine leaves cell suspensions of cv. Gamay treated with laminarin (derived from the brown alga *Laminaria digitata*) 11 defense-related genes were analyzed (Table 2). Six genes were activated rapidly after laminarin treatment (*LOX*, *GST*, *PAL*, *STS1*, *CHIT4c*, and *CHIT1b*), whereas others were up-regulated later (*CHIT3*, *PIN*, *GLU1*, and *PGIP*). This study suggests that laminarin is an efficient elicitor of defense responses in grapevine, as it reduced the development of *B*. *cinerea* and *P*. *viticola* [32]. The exogenous application of abscisic acid in table grapes at different timings (7 or 21 days after veraison) and at different concentrations (200 or 400 mg L^−1^) was evaluated showing an increase in the expression of the anthocyanin biosynthetic genes (*CHI*, *F3’H*, *DFR*, and *UFGT* and of the *VvMYBA1* and *VvMYBA2* transcription factors) after two applications at 400 mg L^−1^ (Table 2) [122]. Modulation of genes related to the flavonoid metabolic pathway (*UFGT*, *OMT2*, *LDOX*, *GST*, *F3’H*, *F3’5’H,* and DFR) has been also reported in cv. Sangiovese vines, subjected to multiple foliar applications of the brown alga *A*. *nodosum* extracts. These genes relative expression was influenced by the growing stage, as well as some defense-related genes (*VvPR1* and *VvCaS2*) were up-regulated [51]. The foliar application of kaolin in grapevine, used as a strategy for summer stress mitigation, triggers metabolic pathways associated with the quality of the berry and, consequently, of the wines [48]. Dinis et al. [48] showed that kaolin exogenous application in cv. Touriga Nacional lowers ROS levels, increases hydroxyl radical scavenging, and enhances the production of antioxidant compounds, including phenolics, flavonoids, and anthocyanins. They showed increases in the transcript abundance of *PAL1* and *CHS1*, genes apparently contributing to the changes in phenolic concentration. In chitosan-treated berries of cvs. Tinto Cão and Touriga Franca ROS pathway genes (*AO*, Fe-*SOD*, *CAT*, *GR*, *Grx*, *Rboh*, *Cu/Zn-SOD*, *POD*, and *PPO*) and the analysis of leaves, stems and shoots revealed that chitosan besides inducing the synthesis of phenolic compounds also acted as a facilitator for transfer of polyphenols from the leaves to the berries [56]. Moreover, in the cv. Tinto Cão, Singh et al. [57] found in chitosan treated leaves and berry skins an up-regulation of several target genes (i.e., *PAL*, *UFGT*, *ABCC1*, *CHS*, *F3H*, *ANR*, *GST*, and *MATE1*) that encode key enzymes and transporters involved in secondary metabolic pathways.

## 6. Conclusions

The worsening of climate change is very problematic for viticulture, increasing the biotic and abiotic stress and consequently compromising the grape quality and yield. Thus, it is urgently necessary to find successful mitigation strategies. In the future, the use of biostimulants will be very important to reduce the use of synthetic chemicals in viticulture. Currently, many biostimulants are being used in grapevines to improve plant physiology and metabolism or act as elicitors. They act as stress alleviators, involved in the resistance against, for example, drought, heat, high irradiation, and also in mechanisms of response to pathogens. In order to use biostimulants with maximum efficiency, it will be important to identify the different mechanisms of action of their bioactive compounds. In addition, the mechanisms triggered by these biostimulants in plants are still poorly understood. There should be a focus on studying different grapevine cultivars and terroirs, as the responses to the application of the same biostimulants may be different depending on the conditions to which the plant is exposed. It will also be important to define the safe concentrations of these biostimulants, the best strategy of application (foliar or other), the number of applications, the phenological stages at which they should be applied, and which biotic or abiotic stress they protect from. Moreover, research on new biostimulant compounds and formulations must be the main goal in order to make viticulture more environment-friendly.

## Figures and Tables

**Table 1:** Application of different biostimulants in grapevine.

Treatment	Matrix	Effect	References
Seaweed Extract	Grape/wine	*A*. *nodosum* extracts, improved quality, increased plant defenses by the regulation of molecular, physiological and biochemical processes.	[49,50,51]
	Grape	*Laminaria digitata* extracts, reduced the infection caused by *P*. *viticola* and *B*. *cinerea*. *Ulva armoricana* extracts protect against *E*. *necator*.	[32,34,52]
Chitosan	Grape	Stimulated defense responses against *P*. *viticola* and *B*. *cinerea*. Increased levels of polyphenols, anthocyanins, and tannins in cv. Tinto Cão berries, and polyphenols and tannins in cv. Touriga Franca. Accumulation of phenolics in berries, namely anthocyanins in cv. Tinto Cão.	[53,54,55,56,57]
	Grapevine	Improved rooting of the cuttings, increased the number of new shoots, their length and number of internodes, as well as the chlorophyll content in the leaves.	[58]
	Grape/wine	The volatile profile (raising the levels of total acetals and alcohols) in cv. Groppello Gentile, responsible for the wine flavor and taste. No substantial effect on phenolic content including anthocyanins, stilbenes, and flavonols, on either grape or wine of cv. Tempranillo.	[17,59]
Yeast Extract	Grape/wine	*Saccharomyces cerevisiae* extracts enhanced anthocyanin and stilbene contents in grape and wine of cv. Tempranillo.	[17]
	Grape	A mixture of *Laminaria digitata* and *Saccharomyces* spp. Extracts used to control *P*. *viticola* in the cv. Montepulciano did not affect grape quality (amino acid content).	[34]
Japanese Knotweed (anthraquinone)	Grape	Protect against *P*. *viticola* in cv. Chasselas.	[42]
Kaolin	Grape	Enhanced photosynthetic activity, improved antioxidant capacity, increased concentration of phenolics including anthocyanins, vitamin C and sugars, in addition to protecting the leaves and berries of cv. Touriga Nacional from solar radiation. Protected the berry exocarp from light stress in cv. Alvarinho.	[2,10,47,48]
Glycine Betaine	Grapevine	Increased the resistance to water stress. Significantly affected leaf relative water content (RWC), canopy temperature, chlorophyll content, soluble carbohydrate contents, and leaf area. Under drought stress, seemed to influence the leaf water status, slightly alleviating the leaf water loss and increasing leaves fresh weight in cv. Alphonse Lavallée.	[60,61]
Methyl Jasmonate	Grapevine	Enhanced tolerance of grapevine foliar cuttings and vineyard against the pathogen *E*. *necator* in cv. Cabernet Sauvignon.	[33]
	Grape/wine	Increased the phenolic compounds content, depending on the variety and vintage.	[17,62,63,64]
Abscisic Acid	Grape	Increased °Brix, total phenolic and anthocyanin contents and improved the production of volatile aromas in cv. Cabernet Sauvignon berries.	[65]
Salicylic Acid	Grape	Increased total phenolic content and antioxidant activity in cv. Syrah. Delayed berries ripening in the cvs. Bez El Naka and Shiraz	[66,67,68]
Others	Grape	Urea: Increased yeast assimilable nitrogen, berry amino acid, flavonoid, stilbene and anthocyanin concentrations. Foliar N and foliar N + S: Improved vine nitrogen status and enhanced aroma expression in Sauvignon blanc wines without the negative impact on vigour and *Botrytis* susceptibility. Phenylalanine: Small improvement in some phenolic compounds.	[69,70,71]

**Table 2:** Genes involved in pathways triggered in grapevine upon application of biostimulants.

Protein (Gene)	Function	References
*Amine oxidase* (*AO*)	ROS pathway gene.	[56]
*Anthocyanin transporters* (*ABCC1*)	Anthocyanin transporter gene.	[57]
*Anthocyanidin reductase* (*ANR*)	Gene of the synthesis pathway of proanthocyanidins.	[57]
*Callose synthase 2* (*VvCaS2*)	Involved in the synthesis of the β-1,3-glucan callose, a plant defense response common to different resistance pathways.	[51]
*Catalase* (*CAT*)	ROS pathway gene.	[56]
*Cu/Zn-superoxide dismutase* (*Cu/Zn-SOD*)	ROS pathway gene.	[56]
*Dihydroflavonol reductase* (*DFR*)	Key gene in the synthesis pathway of anthocyanins.	[51,122]
*Fe superoxide dismutase* (*Fe-SOD)*	ROS pathway gene.	[56]
*Glutathione reductase* (*GR*)	ROS pathway gene.	[56]
*Glutathion-S-transferase (GST)*	Essencial in the detoxification of elicitor-generated oxidants.	[32]
*β-1,3-Glucanase (Glu1)*	Involved in plant defense. Gene encoding a basic β-1,3-glucanase thought to hydrolyse the structural β-1,3-glucan present in some fungal cell walls.	[32,33]
*Leucoanthocyanin Dioxygenase (LDOX)*	Key gene in the synthesis pathway of polyphenols, including anthocyanins.	[2]
*9-Lipoxygenase (LOX)*	Essential for the resistance to fungal infection.	[32]
*MATE1*	Anthocyanin transporter gene.	[57]
*Myeloblastosis A1 and A2 (MYBA1 and MYBA2)*	Key transcription factors of the synthesis pathway of polyphenols, including anthocyanins.	[106,107,123]
*Phenylalanine Ammonia Lyase (PAL)*	Catalyses the first step in the phenylpropanoid pathway.	[2,17,32,106]
*Pathogenesis-Related* (*PR*) *proteins*	Responsible for plant defense by hydrolysing fungal cell wall components. *PR1* is usually reported to be a typical marker of SAR. *PR3*/*PR4*/*PR8*/*PR11* are chitinase proteins, that are strongly induced in the host plant, after the pathogen infection. *PR5* is a thaumatin-like/osmotin, and is a responsive antifungal protein that confers tolerance to both biotic and abiotic stresses in plants. *PR10* may play an important role in the defense of grapevine against *P*. *viticola*. *PR17* is related to defense responses. An accumulation of *PR17* after *P*. *viticola* inoculation has been verified. PIN codifies for a serine-proteinase inhibitor (PIN), a class of antifungal PR-6 proteins, which have potent activity against pathogens.	[32,124,125,126,127]
*Polygalacturonase-inhibiting protein* (*PGIP*)	The PGIP gene product, a polygalacturase-inhibiting protein, interacts with extracellular endo-α-1,4-polygalacturonases (PGs), secreted by phytopathogenic fungi, to inhibit their activity.	[32]
*Resveratrol Synthase* (*STS1*)	Involved in resveratrol and ε-viniferin production, the two major phytoalexinsin the protection against pathogens.	[32]
*UDP-glucose:flavonoid 3-O-glucosyltransferase* (*UFGT*)	Key gene of the flavonoid biosynthetic pathway.	[2,106]

## Data Availability

Not applicable.

## References

[1] Vivier M.A., Pretorius I.S. (2002). Genetically tailored grapevines for the wine industry. Trends Biotechnol..

[2] Conde A., Pimentel D., Neves A., Dinis L.-T., Bernardo S., Correia C.M., Gerós H., Moutinho-Pereira J. (2016). Kaolin foliar application has a stimulatory effect on phenylpropanoid and flavonoid pathways in grape berries. Front. Plant Sci..

[3] Yadav M., Jain S., Bhardwaj A., Nagpal R., Puniya M., Tomar R., Singh V., Parkash O., Prasad G.B.K.S., Marotta F. (2009). Biological and medicinal properties of grapes and their bioactive constituents: An update. J. Med. Food.

[4] Wan Y., Schwaninger H.R., Baldo A.M., Labate J.A., Zhong G.Y., Simon C.J. (2013). A phylogenetic analysis of the grape genus (*vitis l.*) reveals broad reticulation and concurrent diversification during neogene and quaternary climate change. BMC Evol. Biol..

[5] Pilati S., Brazzale D., Guella G., Milli A., Ruberti C., Biasioli F., Zottini M., Moser C. (2014). The onset of grapevine berry ripening is characterized by ROS accumulation and lipoxygenase-mediated membrane oeroxidation in the skin. BMC Plant Biol..

[6] (2021). OIV State of the World Vitivinicultural Sector in 2020.

[7] Shukla P.S., Mantin E.G., Adil M., Bajpai S., Critchley A.T., Prithiviraj B. (2019). *Ascophyllum nodosum*—Based biostimulants: Sustainable applications in agriculture for the stimulation of plant growth, stress tolerance, and disease management. Front. Plant Sci..

[8] Magalhães N., Fer C. (2008). Tratado de Viticultura—A Videira, a Vinha e o “terroir”.

[9] (2010). OIV Definition of Vitivinicultural “Terroir”.

[10] Garrido A., Serôdio J., De Vos R., Conde A., Cunha A. (2019). Influence of foliar kaolin application and irrigation on photosynthetic activity of grape berries. Agronomy.

[11] Jones G., Alves F., Moriondo M., Ferrise R., Santos J., Malheiro A. (2013). Uma Avaliação do Clima para a Região Demarcada do Douro: Uma Análise das Condições Climáticas do Passado, Presente e Futuro Para a Produção de Vinho.

[12] Fraga H., De Cortázar Atauri I.G., Malheiro A.C., Moutinho-Pereira J., Santos J.A. (2017). Viticulture in Portugal: A review of recent trends and climate change projections. OENO One.

[13] Jamiołkowska A. (2020). Natural compounds as elicitors of plant resistance against diseases and new biocontrol strategies. Agronomy.

[14] van Loon L.C., Bakker P.A.H.M., Pieterse C.M.J. (1998). Systemic resistance induced by rhizosphere bacteria. Annu. Rev. Phytopathol..

[15] Kurepin L.V., Ivanov A.G., Zaman M., Pharis R.P., Allakhverdiev S.I., Hurry V., Hüner N.P.A. (2015). Stress-related hormones and glycine betaine interplay in protection of photosynthesis under abiotic stress conditions. Photosynth. Res..

[16] Le Mire G., Nguyen M.L., Fassotte B., Du Jardin P., Verheggen F., Delaplace P., Haissam Jijakli M. (2016). Implementing plant biostimulants and biocontrol strategies in the agroecological management of cultivated ecosystems. A Rev. Biotechnol. Agron. Soc. Environ..

[17] Portu J., López R., Baroja E., Santamaría P., Garde-Cerdán T. (2016). Improvement of grape and wine phenolic content by foliar application to grapevine of three different elicitors: Methyl jasmonate, chitosan, and yeast extract. Food Chem..

[18] Gutiérrez-Gamboa G., Romanazzi G., Garde-Cerdán T., Pérez-Álvarez E.P. (2019). A review of the use of biostimulants in the vineyard for improved grape and wine quality: Effects on prevention of grapevine diseases. J. Sci. Food Agric..

[19] Drobek M., Frąc M., Cybulska J. (2019). Plant biostimulants: Importance of the quality and yield of horticultural crops and the improvement of plant tolerance to abiotic stress-a review. Agronomy.

[20] du Jardin P. (2015). Plant biostimulants: Definition, concept, main categories and regulation. Sci. Hortic..

[21] Gutierrez-Gamboa G., Moreno-Simunovic Y. (2021). Seaweeds in viticulture: A review focused on grape quality. Cienc. E Tec. Vitivinic..

[22] van Leeuwen C., Darriet P. (2016). The impact of climate change on viticulture and wine quality. J. Wine Econ..

[23] Jones G.V., White M.A., Cooper O.R., Storchmann K. (2005). Climate change and global wine quality. Clim. Change.

[24] Fraga H., Malheiro A.C., Moutinho-Pereira J., Santos J.A. (2013). An overview of climate change impacts on European viticulture. Food Energy Secur..

[25] Gambetta J.M., Holzapfel B.P., Stoll M., Friedel M. (2021). Sunburn in grapes: A review. Front. Plant Sci..

[26] Reis S., Fraga H., Carlos C., Silvestre J., Eiras-Dias J., Rodrigues P., Santos J.A. (2020). Grapevine phenology in four Portuguese wine regions: Modeling and predictions. Appl. Sci..

[27] Bulgari R., Franzoni G., Ferrante A. (2019). Biostimulants application in horticultural crops under abiotic stress conditions. Agronomy.

[28] Jones G.V. (2005). Climate change in the western united states grape growing regions. Acta Hortic..

[29] Delaunois B., Farace G., Jeandet P., Clément C., Baillieul F., Dorey S., Cordelier S. (2013). Elicitors as alternative strategy to pesticides in grapevine? Current knowledge on their mode of action from controlled conditions to vineyard. Environ. Sci. Pollut. Res..

[30] Zubrod J.P., Bundschuh M., Arts G., Brühl C.A., Imfeld G., Knäbel A., Payraudeau S., Rasmussen J.J., Rohr J., Scharmüller A. (2019). Fungicides: An overlooked pesticide class? Environ. Sci. Technol..

[31] Wong F.P., Wilcox W.F. (2000). Distribution of baseline sensitivities to azoxystrobin among isolates of *Plasmopara viticola*. Plant Dis..

[32] Aziz A., Poinssot B., Daire X., Adrian M., Bézier A., Lambert B., Pugin A. (2003). Laminarin elicits defense responses in grapevine and induces protection against *Botrytis cinerea* and *Plasmopara viticola*. Mol. Plant-Microbe Interact..

[33] Belhadj A., Saigne C., Telef N., Cluzet S., Bouscaut J., Corio-Costet M.F., Mérillon J.M. (2006). Methyl jasmonate induces defense responses in grapevine and triggers protection against *Erysiphe necator*. J. Agric. Food Chem..

[34] Garde-Cerdán T., Mancini V., Carrasco-Quiroz M., Servili A., Gutiérrez-Gamboa G., Foglia R., Pérez-Álvarez E.P., Romanazzi G. (2017). Chitosan and laminarin as alternatives to copper for *Plasmopara viticola* control: Effect on grape amino acid. J. Agric. Food Chem..

[35] Rantsiou K., Giacosa S., Pugliese M., Englezos V., Ferrocino I., Río Segade S., Monchiero M., Gribaudo I., Gambino G., Gullino M.L. (2020). Impact of chemical and alternative fungicides applied to grapevine cv Nebbiolo on microbial ecology and chemical-physical grape characteristics at harvest. Front. Plant Sci..

[36] Colombo M., Masiero S., Rosa S., Caporali E., Toffolatti S.L., Mizzotti C., Tadini L., Rossi F., Pellegrino S., Musetti R. (2020). NoPv1: A synthetic antimicrobial peptide aptamer targeting the causal agents of grapevine downy mildew and potato late blight. Sci. Rep..

[37] Rouphael Y., Colla G. (2020). Editorial: Biostimulants in Agriculture. Front. Plant Sci..

[38] Bulgari R., Cocetta G., Trivellini A., Vernieri P., Ferrante A. (2015). Biostimulants and crop responses: A review. Biol. Agric. Hortic..

[39] Parađiković N., Teklić T., Zeljković S., Lisjak M., Špoljarević M. (2019). Biostimulants research in some horticultural plant species—A review. Food Energy Secur..

[40] Petit A., Wojnarowiez G., Panon M., Baillieul F., Clément C., Fontaine F., Vaillant-gaveau N. (2009). Botryticides affect grapevine leaf photosynthesis without inducing defense mechanisms. Planta.

[41] Jermini M., Blaise P., Gessler C. (2010). Quantification of the influence of the downy mildew (*Plasmopara viticola*) epidemics on the compensatory capacities of *Vitis vinifera* “Merlot” to limit the qualitative yield damage. Vitis—J. Grapevine Res..

[42] Godard S., Slacanin I., Viret O., Gindro K. (2009). Induction of defence mechanisms in grapevine leaves by emodin- and anthraquinone-rich plant extracts and their conferred resistance to downy mildew. Plant Physiol. Biochem..

[43] Njogu M., Nyankanga R., Muthomi J., Muindi E. (2014). Studies on the effects of stinging nettle extract, phosphoric acid and conventional fungicide combinations on the management of potato late blight and tuber yield in the highlands of Kenya. J. Agric. Food Sci..

[44] Rodino S., Butu M., Butu A. (2018). Alternative antimicrobial formula for plant protection. Bull. USAMV Ser. Agric..

[45] Hildebrandt U., Marsell A., Riederer M. (2018). Direct Effects of physcion, chrysophanol, emodin, and pachybasin on germination and appressorium formation of the barley (*Hordeum vulgare* L.) powdery mildew fungus *Blumeria graminis* f. sp. hordei (DC.). Speer. J. Agric. Food Chem..

[46] Ghazal H.N., Al-Shahwany A.W., Al-Dulaimy F.T. (2019). Control of gray mold on tomato plants by spraying *Piper nigrum* and *Urtica dioica* extracts under greenhouse condition. Iraqi J. Sci..

[47] Dinis L.-T., Ferreira H., Pinto G., Bernardo S., Correia C.M., Moutinho-Pereira J. (2016). Kaolin-based, foliar reflective film protects photosystem II structure and function in grapevine leaves exposed to heat and high solar radiation. Photosynthetica.

[48] Dinis L.T., Bernardo S., Conde A., Pimentel D., Ferreira H., Félix L., Gerós H., Correia C.M., Moutinho-Pereira J. (2016). Kaolin exogenous application boosts antioxidant capacity and phenolic content in berries and leaves of grapevine under summer stress. J. Plant Physiol..

[49] Taskos D., Stamatiadis S., Yvin J.C., Jamois F. (2019). Effects of an *Ascophyllum nodosum* (L.) Le Jol. extract on grapevine yield and berry composition of a Merlot vineyard. Sci. Hortic..

[50] Salvi L., Brunetti C., Cataldo E., Niccolai A., Centritto M., Ferrini F., Mattii G.B. (2019). Effects of *Ascophyllum nodosum* extract on *Vitis vinifera*: Consequences on plant physiology, grape quality and secondary metabolism. Plant Physiol. Biochem..

[51] Frioni T., Tombesi S., Quaglia M., Calderini O., Moretti C., Poni S., Gatti M., Moncalvo A., Sabbatini P., Berrìos J.G. (2019). Metabolic and transcriptional changes associated with the use of *Ascophyllum nodosum* extracts as tools to improve the quality of wine grapes (*Vitis vinifera* cv. Sangiovese) and their tolerance to biotic stress. J. Sci. Food Agric..

[52] Jaulneau V., Lafitte C., Corio-Costet M.F., Stadnik M.J., Salamagne S., Briand X., Esquerré-Tugayé M.T., Dumas B. (2011). An *Ulva armoricana* extract protects plants against three powdery mildew pathogens. Eur. J. Plant Pathol..

[53] Aziz A., Trotel-Aziz P., Dhuicq L., Jeandet P., Couderchet M., Vernet G. (2006). Chitosan oligomers and copper sulfate induce grapevine defense reactions and resistance to gray mold and downy mildew. Phytopathology.

[54] Trotel-Aziz P., Couderchet M., Vernet G., Aziz A. (2006). Chitosan stimulates defense reactions in grapevine leaves and inhibits development of *Botrytis cinerea*. Eur. J. Plant Pathol..

[55] Romanazzi G., Landi L., Feliziani E. (2019). Innovative strategies based on the use of biostimulants to manage plant diseases and minimize the application of synthetic fungicides in grapevine and stone fruits. Med. Jadertina.

[56] Singh R.K., Soares B., Goufo P., Castro I., Cosme F., Pinto-Sintra A., Inês A., Oliveira A., Falco V. (2019). Chitosan upregulates the genes of the ROS pathway and enhances the antioxidant potential of grape (*Vitis vinifera* L. ‘Touriga Franca’ and ’Tinto Cão’) yissues. Antioxidants.

[57] Singh R.K., Martins V., Soares B., Castro I., Falco V. (2020). Chitosan application in vineyards (*Vitis vinifera* L. cv. Tinto Cão) induces accumulation of anthocyanins and other phenolics in berries, mediated by modifications in the transcription of secondary metabolism genes. Int. J. Mol. Sci..

[58] Górnik K., Grzesik M., Romanowska-Duda B. (2008). The Effect of chitosan on rooting of grapevine cuttings and on subsequent plant growth under drought and temperature stress. J. Fruit Ornam. Plant Res..

[59] Vitalini S., Ruggiero A., Rapparini F., Neri L., Tonni M., Iriti M. (2014). The application of chitosan and benzothiadiazole in vineyard (*Vitis vinifera* L. cv Groppello Gentile) changes the aromatic profile and sensory attributes of wine. Food Chem..

[60] Zamani M.M., Rabiei V., Nejatian M.A., Taheri M. (2014). Effect of proline and glycine betaine application on some physiological characteristics in grapevine under drought stress. J. Crop. Improv..

[61] Jalil O.T.J., Sabır A. (2017). Changes in leaf and shoot water statutes of grapevines in response to contrasting water availability and glycine betaine pulverization. Int. J. Agric. Environ. Food Sci..

[62] Gil-Muñoz R., Fernández-Fernández J.I., Crespo-Villegas O., Garde-Cerdán T. (2017). Elicitors used as a tool to increase stilbenes in grapes and wines. Food Res. Int..

[63] Yamaguchi I., Cohen J.D., Culler A.H., Quint M., Slovin J.P., Nakajima M., Yamaguchi S., Sakakibara H., Kuroha T., Hirai N., Liu H.-W., Mander L.B.T.-C.N.P.I.I. (2010). Plant hormones. Comprehensive Natural Products II.

[64] Gil-Muñoz R., Bautista-Ortín A.B., Ruiz-García Y., Fernández-Fernández J.I., Gómez-Plaza E. (2017). Improving phenolic and chromatic characteristics of Monastrell, Merlot and Syrah wines by using methyl jasmonate and benzothiadiazole. OENO One.

[65] Ju Y.L., Liu M., Zhao H., Meng J.F., Fang Y.L. (2016). Effect of exogenous abscisic acid and methyl jasmonate on anthocyanin composition, fatty acids, and volatile compounds of Cabernet Sauvignon (*Vitis vinifera* L.) grape berries. Molecules.

[66] Abdel Salam M. (2016). Effect of foliar application of salicylic acid and micronutrients on the berries quality of “Bez El Naka” local grape cultivar. Middle East J. Appl. Sci..

[67] Blanch G.P., Gómez-Jiménez M.C., del Castillo M.L.R. (2020). Exogenous salicylic acid improves phenolic content and antioxidant activity in table grapes. Plant Foods Hum. Nutr..

[68] Kraeva E., Andary C., Carbonneau A., Deloire A. (1998). Salicylic acid treatment of grape berries retards ripening. Vitis.

[69] Portu J., López R., Santamaría P., Garde-Cerdán T. (2017). Elicitation with methyl jasmonate supported by precursor feeding with phenylalanine: Effect on Garnacha grape phenolic content. Food Chem..

[70] Lacroux F., Trégoat O., Van Leeuwen C., Pons A., Tominaga T., Lavigne-Cruège V., Dubourdieu D. (2008). Effect of foliar nitrogen and sulphur application on aromatic expression of *Vitis vinifera* L. cv. Sauvignon Blanc. OENO One.

[71] Portu J., López-Alfaro I., Gómez-Alonso S., López R., Garde-Cerdán T. (2015). Changes on grape phenolic composition induced by grapevine foliar applications of phenylalanine and urea. Food Chem..

[72] Dhouibi R., Affes H., Ben Salem M., Hammami S., Sahnoun Z., Zeghal K.M., Ksouda K. (2019). Screening of pharmacological uses of *Urtica dioica* and others benefits. Prog. Biophys. Mol. Biol..

[73] Bisht S., Bhandari S., Bisht N.S. (2012). *Urtica dioica* (L): An undervalued, economically important plant. Agric. Sci. Res..

[74] Hadizadeh I., Peivastegan B., Kolahi M. (2009). Antifungal activity of nettle (*Urtica dioica* L.), Colocynth (*Citrullus colocynthis* L. Schrad), Oleander (*Nerium oleander* L.) and Konar (*Ziziphus spina-christi* L.) extracts on plants pathogenic fungi. Pak. J. Biol. Sci..

[75] Guil-Guerrero J.L., Rebolloso-Fuentes M.M., Torija Isasa M.E. (2003). Fatty acids and carotenoids from stinging nettle (*Urtica dioica* L.). J. Food Compos. Anal..

[76] Reeve J.R., Carpenter-Boggs L., Reganold J.P., York A.L., McGourty G., McCloskey L.P. (2005). Soil and winegrape quality in biodynamically and organically managed vineyards. Am. J. Enol. Vitic..

[77] Villanueva-Rey P., Vázquez-Rowe I., Moreira M.T., Feijoo G. (2014). Comparative life cycle assessment in the wine sector: Biodynamic vs. conventional viticulture activities in NW Spain. J. Clean. Prod..

[78] Döring J., Frisch M., Tittmann S., Stoll M., Kauer R. (2015). Growth, yield and fruit quality of grapevines under organic and biodynamic management. PLoS ONE.

[79] Meissner G., Athmann M., Fritz J., Kauer R., Stoll M., Schultz H.R. (2019). Conversion to organic and biodynamic viticultural practices: Impact on soil, grapevine development and grape quality. OENO One.

[80] Tapwal A., Nisha, Garg S., Gautam N., Kumar R. (2011). In vitro antifungal potency of plant extracts against five phytopathogens. Braz. Arch. Biol. Technol..

[81] Anderson H. (2012). Invasive Japanese Knotweed (*Fallopia japonica* (Houtt.)) Best Management Practices in Ontario.

[82] Marchante H., Morais M., Freitas H., Marchante E. (2014). Guia Prático Para a Identificação de Plantas Invasoras em Portugal.

[83] Mahmoud Zaki E.-R., Eid S.Y., Al-Amodi H.S., Wink M. (2016). Fallopia japonica: Bioactive secondary metabolites and molecular mode of anticancer. J. Tradit. Med. Clin. Naturop..

[84] Fouillaud M., Caro Y., Venkatachalam M., Grondin I., Fouillaud M., Caro Y., Venkatachalam M., Grondin I., An L.D., Nollet L.M.L. (2017). Anthraquinones.

[85] Oleszek M., Kowalska I., Oleszek W. (2019). Phytochemicals in Bioenergy Crops.

[86] Barney J.N., Tharayil N., DiTommaso A., Bhowmik P.C. (2006). The Biology of invasive alien plants in Canada. XX. Polygonum cuspidatum Sieb. & Zucc. [=*Fallopia japonica* (Houtt.) Dcne.]. Can. J. Plant Sci..

[87] Patočka J., Navrátilová Z., Ovando M. (2017). Biologically active compounds of Knotweed (*Reynoutria* spp.). Mil. Med. Sci. Lett..

[88] Yang Y.-C., Lim M.Y., Lee H.S. (2003). Emodin isolated from Cassia obtusifolia (Leguminosae) seed shows larvicidal activity against three mosquito species. J. Agric. Food Chem..

[89] Werner S.J., Tupper S.K., Pettit S.E., Ellis J.W., Carlson J.C., Goldade D.A., Hofmann N.M., Jeffrey Homan H., Linz G.M. (2014). Application Strategies for an anthraquinone-based repellent to protect oilseed sunflower crops from pest blackbirds. Crop Prot..

[90] Verkleij F.N. (1992). Seaweed extracts in agriculture and horticulture: A review. Biol. Agric. Hortic..

[91] Zodape S.T., Gupta A., Bhandari S.C., Rawat U.S., Chaudhary D.R., Eswaran K., Chikara J. (2011). Foliar application of seaweed sap as biostimulant for enhancement of yield and quality of tomato (*Lycopersicon esculentum* Mill.). J. Sci. Ind. Res..

[92] Khan W., Rayirath U.P., Subramanian S., Jithesh M.N., Rayorath P., Hodges D.M., Critchley A.T., Craigie J.S., Norrie J., Prithiviraj B. (2009). Seaweed extracts as biostimulants of plant growth and development. J. Plant Growth Regul..

[93] Jayaraman J., Norrie J., Punja Z. (2011). Commercial Extract From the brown seaweed *Ascophyllum nodosum* reduces fungal diseases in greenhouse cucumber. J. Appl. Phycol..

[94] Rayirath P., Benkel B., Mark Hodges D., Allan-Wojtas P., MacKinnon S., Critchley A.T., Prithiviraj B. (2009). Lipophilic components of the brown seaweed, *Ascophyllum nodosum*, enhance freezing tolerance in Arabidopsis thaliana. Planta.

[95] Alam M.Z., Braun G., Norrie J., Hodges D.M. (2012). Effect of *Ascophyllum* extract application on plant growth, fruit yield and soil microbial communities of strawberry. Can. J. Plant Sci..

[96] Correia S., Oliveira I., Queirós F., Ribeiro C., Ferreira L., Luzio A., Silva A.P., Gonçalves B. (2015). Preharvest Application of seaweed based biostimulant reduced cherry (*Prunus avium* L.) cracking. Procedia Environ. Sci..

[97] Jayaraj J., Wan A., Rahman M., Punja Z.K. (2008). Seaweed extract reduces foliar fungal diseases on carrot. Crop Prot..

[98] Correia S., Santos M., Glińska S., Gapińska M., Matos M., Carnide V., Schouten R., Silva A.P., Gonçalves B. (2020). Effects of exogenous compound sprays on cherry cracking: Skin properties and gene expression. J. Sci. Food Agric..

[99] Cabo S., Morais M.C., Aires A., Carvalho R., Pascual-Seva N., Silva A.P., Gonçalves B. (2019). Kaolin and seaweed-based extracts can be used as middle and long-term strategy to mitigate negative effects of climate change in physiological performance of hazelnut tree. J. Agron. Crop Sci..

[100] Cabo S., Aires A., Carvalho R., Vilela A., Pascual-Seva N., Silva A.P., Gonçalves B. (2020). Kaolin, *Ascophyllum nodosum* and salicylic acid mitigate effects of summer stress improving hazelnut quality. J. Sci. Food Agric..

[101] Demain A.L., Phaff H.J., Kurtzman C.P., Cletus P., Kurtzman J.W.F. (1989). The industrial and agricultural significance of yeasts. The Yeasts.

[102] Mukherjee A., Verma J.P., Gaurav A.K., Chouhan G.K., Patel J.S., Hesham A.E. (2020). Yeast a potential bio-agent: Future for plant growth and postharvest disease management for sustainable agriculture. Appl. Microbiol. Biotechnol..

[103] Kapteyn J.C., Van Den E.H., Klis F.M. (1999). The contribution of cell wall proteins to the organization of the yeast cell wall. Biochim. Biophys. Acta.

[104] Gutiérrez-Gamboa G., Portu J., López R., Santamaría P., Garde-Cerdán T. (2018). Elicitor and nitrogen applications to Garnacha, Graciano and Tempranillo vines: Effect on grape amino acid composition. J. Sci. Food Agric..

[105] Gutiérrez-Gamboa G., Portu J., Santamaría P., López R., Garde-Cerdán T. (2017). Effects on grape amino acid concentration through foliar application of three different elicitors. Food Res. Int..

[106] Ferrandino A., Lovisolo C. (2014). Abiotic stress effects on grapevine (*Vitis vinifera* L.): Focus on abscisic acid-mediated consequences on secondary metabolism and berry quality. Environ. Exp. Bot..

[107] Agudelo-Romero P., Erban A., Rego C., Carbonell-Bejerano P., Nascimento T., Sousa L., Martínez-Zapater J.M., Kopka J., Fortes A.M. (2015). Transcriptome and metabolome reprogramming in *Vitis vinifera* cv. Trincadeira berries upon infection with *Botrytis cinerea*. J. Exp. Bot..

[108] Hayat Q., Hayat S., Irfan M., Ahmad A. (2010). Effect of exogenous salicylic acid under changing environment: A review. Environ. Exp. Bot..

[109] Brito C., Dinis L.T., Meijón M., Ferreira H., Pinto G., Moutinho-Pereira J., Correia C. (2018). Salicylic acid modulates olive tree physiological and growth responses to drought and rewatering events in a dose dependent manner. J. Plant Physiol..

[110] Trevisan F., Lima C., Pinto V., Bonome L., de Liz K. (2017). Ácido salicílico no desenvolvimento de plantas e nas características físico-químicas de frutas de morango “Milsei-Tudla”. Rev. Iberoam. Tecnol. Postcosecha.

[111] Dutta T., Neelapu N.R.R., Wani S.H., Challa S., Wani S.H. (2018). Compatible solute engineering of crop plants for improved tolerance toward abiotic stresses. Biochemical, Physiological and Molecular Avenues for Combating Abiotic Stress Tolerance in Plants.

[112] Adak N. (2019). Effects of glycine betaine concentrations on the agronomic characteristics of strawberry grown under deficit irrigation conditions. Appl. Ecol. Environ. Res..

[113] Mickelbart M.V., Chapman P., Collier-Christian L. (2006). Endogenous levels and exogenous application of glycine betaine to grapevines. Sci. Hortic..

[114] Hussain Wani S., Brajendra Singh N., Haribhushan A., Iqbal Mir J. (2013). Compatible solute engineering in plants for abiotic stress tolerance—Role of glycine betaine. Curr. Genom..

[115] Hayes M.A., Shor A.C., Jesse A., Miller C., Kennedy J.P., Feller I. (2020). The role of glycine betaine in range expansions; protecting mangroves against extreme freeze events. J. Ecol..

[116] Awad M.A., Al-Qurashi A.D., Mohamed S.A. (2015). Postharvest trans-resveratrol and glycine betaine treatments affect quality, antioxidant capacity, antioxidant compounds and enzymes activities of ‘El-Bayadi’ table grapes after storage and shelf life. Sci. Hortic..

[117] Tisarum R., Theerawitaya C., Samphumphuang T., Singh H.P., Cha-um S. (2020). Foliar application of glycine betaine regulates soluble sugars and modulates physiological adaptations in sweet potato (*Ipomoea batatas*) under water deficit. Protoplasma.

[118] Khadouri H.K., Kandhan K., Salem M.A. (2020). Effects of glycine betaine on plant growth and performance of *Medicago sativa* and *Vigna unguiculata* under water deficit conditions. J. Phytol..

[119] Correia S., Queirós F., Ribeiro C., Vilela A., Aires A., Barros A.I., Schouten R., Silva A.P., Gonçalves B. (2019). Effects of calcium and growth regulators on sweet cherry (*Prunus avium* L.) quality and sensory attributes at harvest. Sci. Hortic..

[120] Rouphael Y., Carillo P., Colla G., Fiorentino N., Sabatino L., El-Nakhel C., Giordano M., Pannico A., Cirillo V., Shabani E. (2020). Appraisal of combined applications of trichoderma virens and a biopolymer-based biostimulant on lettuce agronomical, physiological, and qualitative properties under variable n regimes. Agronomy.

[121] Nardi S., Carletti P., Pizzeghello D., Muscolo A., Senesi N., Xing B., Huang P. (2009). Biological activities of humic substances. Biophysico-Chemical Processes Involving Natural Nonliving Organic Matter in Environmental Systems.

[122] Koyama R., Roberto S.R., de Souza R.T., Borges W.F.S., Anderson M., Waterhouse A.L., Cantu D., Fidelibus M.W., Blanco-Ulate B. (2018). Exogenous abscisic acid promotes anthocyanin biosynthesis and increased expression of flavonoid synthesis genes in *Vitis vinifera* × *Vitis labrusca* table grapes in a subtropical region. Front. Plant Sci..

[123] Dong T., Zheng T., Fu W., Guan L., Jia H., Fang J. (2020). The effect of ethylene on the color change and resistance to *Botrytis cinerea* infection in “Kyoho” grape fruits. Foods.

[124] Legay G., Marouf E., Berger D., Neuhaus J.M., Mauch-Mani B., Slaughter A. (2011). Identification of genes expressed during the compatible interaction of grapevine with *Plasmopara viticola* through suppression subtractive hybridization (SSH). Eur. J. Plant Pathol..

[125] Kumar S.A., Kumari P.H., Kumar G.S., Mohanalatha C., Kishor P.B.K. (2015). Osmotin: A plant sentinel and a possible agonist of mammalian adiponectin. Front. Plant Sci..

[126] Dufour M.C., Magnin N., Dumas B., Vergnes S., Corio-Costet M.F. (2016). High-throughput gene-expression quantification of grapevine defense responses in the field using microfluidic dynamic arrays. BMC Genomics.

[127] Nesler A., Perazzolli M., Puopolo G., Giovannini O., Elad Y., Pertot I. (2015). A complex protein derivative acts as biogenic elicitor of grapevine resistance against powdery mildew under field conditions. Front. Plant Sci..

